# Vitrectomy With Peeling the Internal Limiting Membrane for the Treatment of Macular Hole Following Ruptured Retinal Arterial Macroaneurysm: A Case Report

**DOI:** 10.3389/fmed.2021.793054

**Published:** 2021-12-24

**Authors:** Zhigao Liu, Shuya Wang, Yu Wang, Aihua Ma, Bojun Zhao

**Affiliations:** ^1^First Clinical Medical College, Shandong University of Traditional Chinese Medicine, Jinan, China; ^2^Department of Ophthalmology, Jinan Aier Eye Hospital, Jinan, China; ^3^Department of Pediatrics, Shandong Provincial Hospital Affiliated to Shandong First Medical University, Jinan, China; ^4^Department of Ophthalmology, Shandong Provincial Hospital Affiliated to Shandong First Medical University, Jinan, China

**Keywords:** retinal arterial macroaneurysm, macular hole (MH), vitrectomy, visual acuity, ILM peeling

## Abstract

**Background:** This study aimed to report a case of vitrectomy with peeling the internal limiting membrane for the treatment of macular hole (MH) following ruptured retinal arterial macroaneurysm (RAMA).

**Case Presentation:** A 65-year-old woman noticed a sudden decrease in vision in the left eye. She had no other ocular problems apart from a mild cataract in both eyes before. Her best-corrected visual acuity (BCVA) was 20/33 in the right eye, and 6/100 in the left eye. Fluorescein angiography (FFA) showed a retinal arterial macroaneurysm with telangiectatic retinal vascular changes in the inferior temporal macular region. Optical coherence tomography (OCT) examination demonstrated the presence of subretinal hemorrhage extending into the foveal area and incomplete posterior vitreous detachment. Because of the presence of submacular hemorrhage, some medicine was administrated and the patient was followed up. Then, 5 months later, the hemorrhage was absorbed. OCT examination exhibited a full-thickness MH with a macular epiretinal membrane. The size of the MH was 722 μm in diameter. She was then given a standard three-port pars plana vitrectomy (PPV), along with peeling of the internal limiting membrane (ILM) and filling the vitreous cavity with air. Anatomic closure of the MH was achieved after 4 weeks of the surgery by the examination of OCT. The BCVA was improved to 15/100.

**Conclusions:** This case expanded our knowledge of the association of MH secondary to ruptured RAMA. We reported a case with successful surgical closure of the MH and improvement of BCVA.

## Background

Macular holes (MHs) are usually idiopathic and occur most commonly in healthy, middle-aged women. In rare cases, MHs have been associated with retinal vascular diseases such as proliferative diabetic retinopathy (1), hypertensive retinopathy (2), congenital retinal arteriovenous communication (3), and retinal arterial macroaneurysm (4). The onset of MH after the rupture of a retinal arterial macroaneurysm (RAMA) is a sight-threatening complication (5, 6). Previous reports have suggested that 5.9–12.5% of RAMA ruptures result in MH and that anatomic successful rate of surgical intervention for MH secondary to RAMA is between 57.1 and 75%(7, 8). Which is lower than that of surgical intervention for idiopathic MH (9). Moreover, there are limited effective surgical procedures for the treatment of MH after RAMA rupture.

Several studies have reported the efficacy of autologous transplantation of ILM (10, 11) and the inverted ILM flap technique (12) for the treatment of MH. In our report, we described the application of vitrectomy with peeling the Internal limiting membrane for the treatment of stage IV MH caused by RAMA rupture.

## Case Presentation

**Figure 1:**
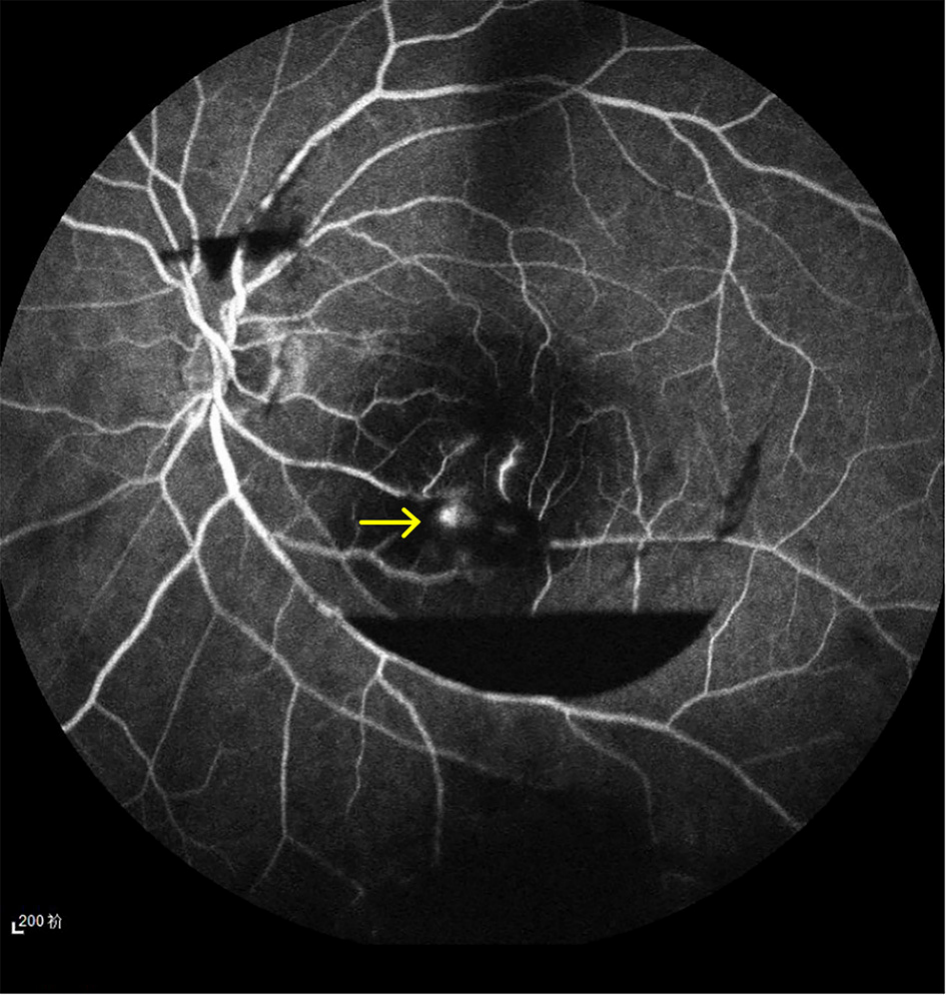
A retinal arterial macroaneurysm with telangiectatic retinal vascular changes in the inferior temporal macular region (yellow arrow shows).

**Figure 2:**
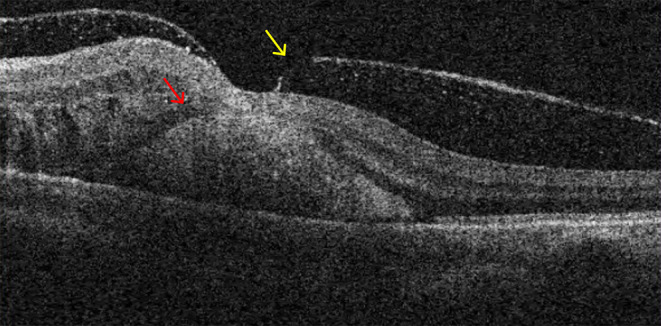
The presence of subretinal hemorrhage which extending into the foveal area (red arrow shows) and incomplete posterior vitreous detachment (yellow arrow shows).

**Figure 3:**
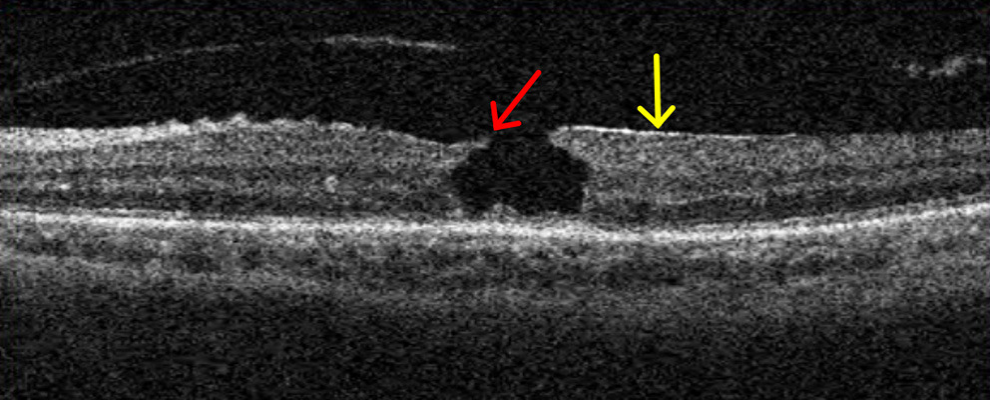
A full-thickness macular hole (MH) (red arrow shows) with macular epiretinal membrane (yellow arrow shows).

**Figure 4:**
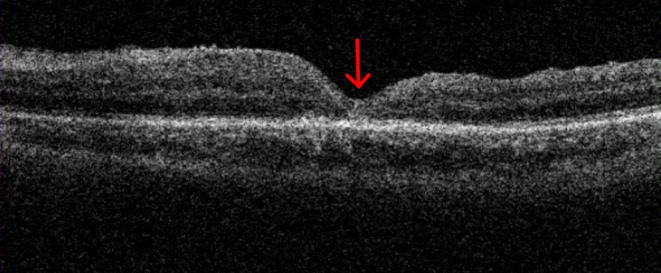
Anatomic closure of the MH (red arrow shows) was achieved after 4 weeks of the surgery. The BCVA was improved to 15/100.

A 65-year-old woman with a longstanding history of hypertension noticed a sudden decrease in vision in her left eye. She had no other ocular problems apart from a mild cataract in both eyes before. Her best-corrected visual acuity was 20/33 in her right eye, and 6/100 in her left eye. Fluorescein angiography showed a retinal arterial macroaneurysm with telangiectatic retinal vascular changes in the inferior temporal macular region (Figure 1). Optical coherence tomography (OCT) examination demonstrated the presence of subretinal hemorrhage which extends into the foveal area, and incomplete posterior vitreous detachment (Figure 2). Because of the presence of submacular hemorrhage, some medicine was administrated and the patient was followed up. Then, 5 months later, the hemorrhage was absorbed. OCT examination exhibited a full-thickness MH with a macular epiretinal membrane (Figure 3). The size of the MH was 722 μm in diameter. She was then given a standard three-port pars plana vitrectomy, along with peeling of the internal limiting membrane and filling the vitreous cavity with air. Anatomic closure of the MH was achieved after 4 weeks of the surgery by the examination of OCT (Figure 4). The BCVA was improved to 15/100.

Pars plana vitrectomy (PPV) method:

Standard three-port vitrectomy was performed, followed by surgical separation of the posterior cortical vitreous from the optic nerve and posterior retina. About 1% indocyanine green (ICG) was injected over the macula region after temporarily stopping the infusion. The ILM with 2 disk diameters of the fovea was removed. The vitreous cavity was filled with air. The patient was asked to remain face down for a week following the surgery.

## Discussion

The formation of MH following a ruptured RAMA is not entirely uncommon. Colucciello and Nachbar (5) reported a 67-year-old female patient who developed a full-thickness MH at 5 months after being diagnosed with a ruptured RAMA. A thickened, taut posterior vitreous cortex was observed during vitrectomy. Ciardella and Gaetano (13) reported a patient who developed a stage IV MH in association with a retinal arterial macroaneurysm combined with a total posterior vitreous detachment. In our case, a possible reason for the formation of the MH was that a posterior vitreous detachment induced by the submacular hemorrhage. Another possibility was that acute subretinal hemorrhage might elevate the pressure in the subretinal space which led to the dehiscence of the fovea. The anatomic successful rate of surgical intervention for MH secondary to RAMA is lower than that of idiopathic MH (4, 8, 9). Thus, it is very important to explore surgical methods for the treatment of MH secondary to RAMA. Kumagai et al. (14) reported a retrospective, non-randomized, comparative trial which showed that Internal limiting membrane peeling in MH surgery achieved high closure and low reopening rates. Brooks (9) reported that ILM peeling significantly improved vision and anatomic recovery in all stages of recent and chronic MHs.

In conclusion, this case expanded our knowledge of the association of MHs secondary to ruptured RAMA. We reported a case with successful surgical closure of the MH and improvement of BCVA. Large-scale, randomized, comparative, and prospective studies are needed to further warrant this method.

## Data Availability Statement

The original contributions presented in the study are included in the article/supplementary material, further inquiries can be directed to the corresponding author/s.

## Ethics Statement

The studies involving human participants were reviewed and approved by the Ethics Committee of Jinan Aier Eye Hospital. The patients/participants provided their written informed consent to participate in this study. Written informed consent was obtained from the individual(s) for the publication of any potentially identifiable images or data included in this article.

## Author Contributions

ZL contributed to writing the manuscript. YW and SW contributed to the literature research and preparation of the manuscript and figures. AM and BZ are responsible for the design of the case report. All authors read and approved the final manuscript.

## Funding

This work was supported by Shandong Natural Foundation, China to BZ and AM (ZR2019MH111 and ZR2017MH021).

## Conflict of Interest

The authors declare that the research was conducted in the absence of any commercial or financial relationships that could be construed as a potential conflict of interest.

## Publisher's Note

All claims expressed in this article are solely those of the authors and do not necessarily represent those of their affiliated organizations, or those of the publisher, the editors and the reviewers. Any product that may be evaluated in this article, or claim that may be made by its manufacturer, is not guaranteed or endorsed by the publisher.

## Abbreviations

MH: macular holes
RAMA: rupture of a retinal arterial macroaneurysm.
